# Effects of late incubation temperature and moment of first post-hatch feed access on neonatal broiler development, temperature preference, and stress response

**DOI:** 10.1016/j.psj.2022.102088

**Published:** 2022-08-03

**Authors:** H.J. Wijnen, R. Molenaar, B. Kemp, I.A.M. van Roovert-Reijrink, H. van den Brand, C.W. van der Pol

**Affiliations:** ⁎ Research Department, Hatchtech BV, Veenendaal, the Netherlands; † Adaptation Physiology Group, Department of Animal Sciences, Wageningen University and Research, Wageningen, the Netherlands

**Keywords:** broiler chicken, eggshell temperature, early feeding, stress, temperature preference

## Abstract

Early life experiences are known to be of great importance for later life. For instance, exposure to stress during early life can increase fearfulness at later age. In broilers, delayed feeding after hatch may cause metabolic stress. Besides, delayed feeding after hatch may affect neonatal broiler development and thermogenesis and consequently preferred ambient temperature. Moreover, these effects of feeding strategy may be dependent on late incubation temperature. To study this, eggs (n = 1,338) from a 54-wk-old Ross broiler breeder flock were incubated at 37.8°C (**control**) or 36.7°C (**lower**) eggshell temperature (**EST**) during late incubation (≥ embryonic d 17). At hatch, two feeding strategies were applied (direct access (**early feeding**) or 51 to 54 h delayed access (**delayed feeding**)). Broilers (n = 960) were equally divided over 32 pens and grown for 3 wk. Stress was assessed by determination of corticosterone in blood at 0 h, 48 h, 96 h and d 21 after hatch. Fearfulness was assessed by tonic immobility at d 13. Temperature preference was assessed at d 2 and d 12. Broiler development was determined at 0 h, 48 h, and 96 h after hatch. There was no EST × feeding strategy interaction for any parameter (*P* ≥ 0.07). Early feeding resulted in a 2.5× lower plasma corticosterone concentration at 48 h (*P* < 0.01) and a 2.2°C and 2.0°C lower preference temperature for d 2 and d 12 respectively (*P* = 0.01) compared to delayed feeding. Tonic immobility was not affected. In conclusion, early feeding reduces exposure to stress in the short term and stimulates thermoregulatory ability of broilers in the longer term.

## INTRODUCTION

The perinatal period of animals is known to be of great importance for their later health, welfare, and performance. This period in life contains critical windows during which the phenotype can be shaped permanently by environmental factors. Such alterations in phenotype during early life can determine animal behavior during environmental challenges in later life. For example, in laying hens it was shown that injection of corticosterone in eggs, as a model for exposure to prenatal stress, resulted in higher fear responses of hens at 2 wk post hatch (Janczak et al., 2006).

One event during the perinatal life of poultry that may cause stress is delayed access to feed and water after hatch moment (referred to as “**delayed feeding**”). For practical reasons, most chicks in commercial practice are withheld from feed and water during the period from hatch at the hatchery until placement at the farm. Withholding or partial restriction of feed and/or water for 4 h or longer has been shown to cause a stress reaction in chickens aged 3 wk or older, indicated by higher plasma corticosterone concentrations, increased peripheral heterophil to lymphocyte ratio, and/or behavioral changes (Buckland et al., 1974; Nir et al., 1975; Freeman et al., 1980; Scott et al., 1983; Hocking et al., 1996; Najafi et al., 2015; Pál et al., 2015). However, studies that investigated whether delayed feeding causes stress in neonatal chicks are limited (Gonzales et al., 2003; van de Ven et al., 2013; Khosravinia and Manafi, 2016; Shakeel et al., 2016) and their results are inconsistent (reviewed by de Jong et al., 2017). Moreover, possible effects of neonatal stress due to delayed feeding on stress response at later age are unknown.

Besides stress mechanisms, delayed feeding may evoke its effects through other physiological mechanisms. For instance, it was shown that providing feed and water directly after hatch (referred to as ‘**early feeding**’; Roberts, 1928) increased metabolic heat production and improved the resistance of neonatal chicks against cold exposure (Decuypere and Kuhn, 1988; van den Brand et al., 2010; van Roovert-Reijrink et al., 2017). This may suggest that early fed broilers may prefer a lower ambient temperature compared to delayed fed broilers during grow out, but this has not been studied yet. Apart from early feeding, incubation temperature may affect preferred ambient temperature as well. For example, a higher incubation temperature from d 13 of incubation until hatching resulted in a ~ 1.5°C higher preferred ambient temperature up to 15 d post hatch (Morita et al., 2016a,b). A lower incubation temperature during the last week of incubation tended to result also in a 1.4°C higher preferred ambient temperature at d 1 post hatch (Wijnen et al., 2020). Thus, early fed chickens may prefer a lower ambient temperature during rearing compared to delayed fed chickens when late incubation temperature was standard, whereas this may not be true at higher or lower late incubation temperatures.

De Jong et al. (2017) suggested that effects of early feeding may indeed be dependent on incubation temperature. The authors showed that early feeding can affect mortality rate and neonatal body development in terms of organ weights, gut development, immune response, and residual yolk (**RY**) uptake. However, considerable variation and inconsistency among studies existed and these ambiguous results might be related to, among other factors, incubation temperature. Incubation temperature is known to have a major impact on embryo development and consequently chick quality at hatch. Currently, a constant eggshell temperature (**EST**) of 37.8°C throughout incubation is regarded optimal (Lourens et al., 2005). However, a lower EST of 36.7°C during the last week of incubation was shown to increase yolk-free body mass (**YFBM**) and heart weight at hatch (Maatjens et al., 2016; Wijnen et al., 2020). Newly hatched chicks with a higher YFBM have relatively less RY available that could be used for body development during the neonatal period. Therefore, the importance of early feed intake may be higher for these chicks.

We hypothesize that delayed feeding will be stressful for the neonatal chick, leading to elevated corticosterone levels in the short term and resulting in higher fearfulness at a later age; delayed feeding results in higher preferred ambient temperatures during rearing; and that late incubation temperature interacts with post-hatch feeding strategy on temperature preference and neonatal chick development.

## MATERIALS AND METHODS

An experiment was set up as a 2 × 2 factorial arrangement with 2 EST's during late incubation and two feeding strategies after hatch. From embryonic d (**E**) 17 until E19 h12 EST was set either at 37.8°C (**control**) or at 36.7°C (**lower**), whereas feeding strategy was either access to feed and water within 3 to 6 h after hatch (**early feeding**) or within 51 to 54 h after hatch (**delayed feeding**). The experimental protocol was approved by the Governmental Commission on Animal Experiment, The Hague, the Netherlands, approval number: 2018.W-0020.001 and by the Ethical Committee of Poulpharm, Belgium, approval number P19034-FP. A brief description of material and methods is provided in this section. For more details please see Wijnen et al. (2021).

### Incubation

In total 1,338 eggs from a 54-wk-old Ross 308 broiler breeder flock were divided over 16 setter trays (type 88 Setter Tray, HatchTech, Veenendaal, the Netherlands). All 16 trays were set in one climate respiration chamber, which was used as incubator (details provided by Heetkamp et al., 2015) in which 4 EST sensors (NTC Thermistors: type DC 95; Thermometrics, Somerset, UK) were each attached to an individual egg, equally divided over the incubator. Incubator temperature was continuously adjusted, based on the median temperature of these 4 EST sensors to aim at an EST of 37.8°C up to E17. RH was maintained between 50 and 55%, CO_2_ level was maintained below 3,500 ppm, and eggs were turned every h by an angle of 45° from horizontal.

At E17, all eggs were candled and eggs containing a viable embryo (86.7% of fertile eggs at set) were transferred to one hatching basket per setter tray. Hatching baskets were divided over 4 incubators (4 baskets / setter) in which EST control was performed as described above. Two incubators were set at an EST of 37.8°C, whereas the other 2 incubators were set at an EST of 36.7°C. RH was maintained between 45 and 75% and CO_2_ levels were maintained below 3,500 ppm. At 468 h after the onset of incubation (E19 12 h), the air temperatures in the incubators were fixed at their actual temperature setting and EST was allowed to change.

### Hatch and Early Feeding

From 468 h of incubation until the last chick hatched (E21 0 h), the incubators were opened every 3 h to check whether or not chicks had hatched. Any newly hatched chick was marked, put back in the original hatching basket in the incubator and left to dry for 3 h. After 3 h, the chick was pulled from the incubator and classified either as 1st grade chick (no abnormalities) or 2nd grade chick if any abnormality was observed (e.g., crossed beak, blindness, exposed brains, extra legs, exposed yolk). Any 1st grade chick (n = 1,028) was feather sexed, received a unique neck tag, and was transferred to a HatchCare basket (HatchTech, Veenendaal, the Netherlands) that was placed in a chick storage room at 36.0°C and 55% RH until 516 h after the onset of incubation (E21 12 h). In the chick storage room, half of the baskets were provided ad libitum with fresh water and starter pellet feed (early feeding treatment groups) whereas no feed and water was provided in the other half of the baskets (delayed feeding). At 516 h after the onset of incubation, all chicks were transported in a climate-controlled van (29.4°C and 36% RH) for approximately 3 h to a grow-out facility in Zwevegem, Belgium.

### Grow-out

#### Housing

Chicks were divided over 32 floor pens in one broiler house with 30 broilers (15 male and 15 female) per pen and grown for 3 wk. Each pen contained chicks that hatched equally distributed over the hatch window and from all hatching baskets of the corresponding treatment group. Pens were divided over 8 equal blocks and each block contained all 4 treatment groups (2 EST × 2 feeding strategies). Pen size was 260 × 105 cm. The floor was covered with wood shavings and within each pen 4 drinking nipples and a feed silo were provided. House temperature setpoint was 35°C at placement and was linearly decreased to 22.5°C at d 21. A heating lamp was provided in the middle of each pen from placement until d 12, meaning that broilers were able to choose their own preferred ambient temperature. RH was on average 34.5% ± 15%. Continuous light was provided until d 2 and thereafter 1 h of darkness / 24 h was added each d until 18L:6D was provided by d 7 to d 21.

#### Diet and Vaccinations

At placement the pens from the delayed feeding treatment were divided with a fence in the middle into an unfed side and a fed side. The litter of the unfed side was covered with cardboard to prevent litter consumption. At placement, all delayed fed broilers were positioned in the unfed side of the pen and each broiler was relocated individually to the fed side of the pen 48 h after it had received its neck tag. Fences and cardboard were removed after all broilers were relocated to the fed side of the pen.

A starter pelleted diet was provided from hatch until d 13 and a grower pelleted diet from d 13 until d 21. Both diets did not contain coccidiostats and were provided ad libitum. The grower diet included fish meal (10%) and rye (5%) as predisposing factors for the development of necrotic enteritis after d 21 (see Wijnen et al., 2021). Newcastle disease (Avishield ND) and infectious bronchitis (Poulvac IB primer) spray vaccinations were administered at placement. At d 12, Newcastle disease (Avishield ND) and Gumboro disease (Nobilis Gumboro D78) vaccinations were provided via drinking water.

### Data Collection

All 16 setter trays were bulk-weighed at set and at E17, prior to the onset of EST treatments, to calculate the average egg weight loss. Eggs candled out at E17 were opened and scored either as infertile or as dead embryo. Actual EST from all eggshell sensors was logged every minute. For each sensor, average EST per h was calculated. These averages were used to calculate average, minimum, and maximum EST per h per treatment. Effects of EST on incubation and embryo development were studied by determining hatchability, hatch window, incubation duration, and chick quality at hatch. Hatchability was calculated per hatching basket as the number of hatched chicks divided by the number of eggs that contained a viable embryo at E17. Incubation duration was calculated as the number of h from E0 (start of incubation) to emergence from the eggshell. Hatch window was calculated as incubation duration of the last chick minus incubation duration of the first chick.

Chick quality of 1st grade chicks was determined by measuring BW, chick length, and navel score as described by Wijnen et al. (2020). Additionally, every twelfth chick per treatment that hatched was euthanized through decapitation until 25 chicks per treatment were collected. These chicks were opened and RY, heart, intestines, bursa, and stomach were removed and weighed on a 3-decimal scale. Stomach included the proventriculus, the intermediate zone, the ventriculus, and the pylorus. YFBM was calculated as BW minus RY weight. Relative organ weights were calculated as percentage of YFBM. After weighing the intestines, approximately 1.5 cm of the jejunum was collected (0.5 cm posterior the distal part of the duodenum). A Swiss roll was made as described by Moolenbeek and Ruitenberg (1981), fixated in 4% formaldehyde in PBS for 2 d, and stored in 70% ethanol until processing. At processing, tissues were embedded in paraffin, 3-µm sliced, mounted on a glass microscope slide, and hematoxylin and eosin stained. From 12 random chicks per treatment, one slide was analyzed on a microscope (Leica DM3000 LED, LAS V4.9-software) for villi and crypt appearance by a blinded observer. From each slide, 5 to 10 villi and associated crypts were randomly chosen. Villi lengths and crypt depths were determined as described by Uni et al. (1995) and the averages of these 5 to 10 villi and crypts per slide were calculated and used for statistical analysis. Villi: crypt ratio (**V:C**) was calculated as villi length divided by crypt depth for each individual villi and associated crypt, whereafter the averages were used for statistical analysis.

After decapitation at hatch moment, blood was collected in serum tubes (Vacuette 5 mL, Greiner Bio-One, Alphen aan de Rijn, the Netherlands), stored on ice, and serum was collected after centrifugation at 5,251 × *g* for 10 min at 4°C. Serum was stored at -20°C until samples were analyzed for natural antibodies against keyhole limpet hemocyanin (**NAb**) isotypes IgM and IgY according to an adjusted protocol from Lammers et al. (2004), described by Wijnen et al. (2021). Titers were calculated according to the protocol from Van der Klein et al. (2015).

Neonatal broiler development was determined at 48 and 96 h after hatch. At both ages, 1 female and male broiler was selected randomly per pen with Excel randomizer. Selected chicks were weighed and then euthanized by decapitation. RY, YFBM, relative bursa and heart weight, villi and crypt morphology, and IgY and IgM NAb were determined as described in the previous paragraph. NAb IgM isotype was not found in serum of any broiler and will therefore not be discussed any further. Mortality and culled broilers (humane end points as defined by Marchewka et al., 2013) were recorded daily during the 1st wk. Mortality percentage was calculated per pen by summing up the number of broilers that died and were culled as percentage of broilers at placement minus the number of broilers that were dissected.

Temperature preference was determined at d 2 (4 pens per treatment group) and d 12 (5 pens per treatment group) by observing behavior during a temperature preference test that was adapted from the protocol of Walstra et al. (2010). Two male and 2 female broilers per pen were randomly picked up and were placed together in the middle of a test box which was situated in a room adjacent to the broiler house. This wooden box (160 × 60 × 50 cm) was covered with a Plexiglas lid, had wood shavings at the bottom, and 2 infrared heat bulbs (250 Watt each) situated at one side of the box such that a linear temperature gradient from 20°C to 50°C was created. Temperature sensors at broiler height (NTC DC95 thermistors, Thermometrics, Somerset, UK) continuously monitored the actual temperature at 24 locations in the box equally spread over the total gradient. Generally, broilers laid down and remained on the same position from approximately 15 min after placement onwards. Therefore, 20 min after placement in the box, the location of each broiler was noted as well as the associated actual sensor temperature. From d 1 to d 7, in all pens cloacal temperature (VT1831, Microlife, Widnau, Switzerland) was determined each morning in the same 3 broilers per pen (at least 1 broiler of both sexes was included).

Stress responsiveness was studied by measuring corticosterone, and tonic immobility. Corticosterone was analyzed in serum of all broilers that were dissected at hatch, 48 h after hatch, and 96 h after hatch. Additionally, corticosterone was analyzed in serum collected at d 21 from in total 100 broilers (details provided in Wijnen et al., 2021). Corticosterone was analyzed in duplo in all serum samples. Serum was diluted 5× with steroid diluent from a radioimmunoassay kit (RIA A68390, Beckman Coulter, Indianapolis, IN) and subsequently analyzed with this kit according to the protocol from Van der Pol et al. (2019). Tonic immobility was assessed at d 13. One male and female broiler per pen were randomly picked up and the latency until the broiler was attempting to stand up as well as the number of vocalizations and the number of attempts to put a broiler on the back were assessed according to a protocol adapted from Jones (1986). A broiler was transported by hand from its home pen to a table in the corner of the broiler house. Two observers were standing next to the table without making eye contact with the broiler at any time during the test. One and the same observer induced tonic immobility by gently laying the broiler on its back on the table and consequently holding one hand on the sternum and lightly covering the eyes with the other hand during 10 s. After 10 s, the observer slowly removed both hands and meanwhile the other observer started recording. If the broiler stood up within 10 s after removing hands, the procedure was repeated up to a maximum of 5 times. After 5 unsuccessful attempts, the test was stopped, and another pen mate was selected randomly. Latency until attempt to stand up was recorded once a broiler remained down for at least 10 s after removing hands. If a broiler did not stand up within 600 s, the test was stopped and a latency of 600 s was noted.

### Statistical Analyses

Data was analyzed with the statistical software package SAS (Version 9.4, SAS institute, 2010). The basic model used for all data at hatch was

(1) $$Y_{i}=\mu +\text{ES}T_{i}+e_{i},$$

where, Y_i_ = the dependent variable, µ = the overall mean, EST_i_ = eggshell temperature during late incubation (_i_ = 36.7°C or 37.8°C), and e_ij_ = the error term. To analyze hatchability, hatching basket was considered to be the experimental unit and incubator was added as a random factor to model 1. All other data at hatch was collected for individual chicks, but hatching basket was considered to be the experimental unit by extending model 1 with hatching basket nested within incubator as a random factor (CRD). Sex was added to model 1 as a fixed factor. Preliminary statistical analysis did not show significant effects of EST × sex for any of the variables, except for navel score. Therefore, interactions between sex and treatments were excluded from the final model with the exception of navel score. For chick length, the ID code of the person that performed the measurement was added to the model as a fixed factor.

To analyse data that was collected after hatch, model 1 was extended with feeding strategy and sex as follows;

(2) $$Y_{\text{ij}}=\mu +\text{ES}T_{i}+\text{FEE}D_{j}+\text{se}x_{k}+\text{interactions}+e_{\text{ijk}},$$

where FEED_j_ = feeding strategy (_j_ = early or delayed), sex_k_ = sex (k = male or female), interactions = 2 and 3-way interactions between EST, FEED, and sex, and e_ijk_ = the error term. Preliminary statistical analysis did not show significant effects of 3-way interactions, sex × EST, or sex × FEED for any of the variables. Therefore, these interactions were excluded from the final model. All data after hatch was collected for individual broilers, but pen was considered to be the experimental unit by extending model 2 with pen as a random factor with the exception of mortality (CRD). Mortality was collected per pen and therefore no random factor was added and sex was excluded from model 2.

The PROC MIXED procedure was used to analyze all data, except for navel score, number of vocalisations, and number of back attempts during tonic immobility. Model assumptions were verified by inspection of residual plots and not normally distributed data were log transformed. Data is expressed as least square mean ± SEM. Tukey adjustments for multiple comparisons were used to compare least square means. A *P*-value ≤ 0.05 was considered to be significant and a *P*-value > 0.05 and ≤ 0.10 as a tendency.

The PROC GLIMMIX procedure was used to analyze navel score, number of vocalizations, and number of back attempts during tonic immobility. For navel quality score navel scores 2 and 3 were grouped and analysed as binary data, using a logit link function in model 1. Number of vocalisations and induction attempts during tonic immobility were analyzed with a Poisson log link function in model 2. Data is expressed as mean ± SE. A *P*-value ≤ 0.05 was considered to be significant and a *P*-value > 0.05 and ≤ 0.10 as a tendency.

## RESULTS

### Incubation

Egg weight loss between set and E17 was on average 8.6%. Infertility and embryonic mortality of set eggs were 4.6 and 1.8%, respectively. Average, minimum, and maximum EST per hour per treatment between E17 and E19 h12 are provided as supplementary data (Figure S1). No effect of EST on hatchability (*P* = 0.26; average 97.2% ± 1.2) nor on duration of the hatch window (37 h for both EST treatments) was found. Incubation duration was on average 7 h longer for lower EST compared to control EST (*P* < 0.001; Table 1).

**Table 1:** Effect of eggshell temperature (EST) during late incubation (≥ embryonic d 17; 37.8°C [**control**] or 36.7°C [**lower**]) on chick quality characteristics at hatch (LSmean ± SEM)

n^1^	Duration (h)	BW (g)	RY^2^ (g)	YFBM^2^ (g)	Length (cm)	Heart (%)^3^	Bursa (%)^3^	NAb^2^ (titre)	Stomach^2^ (%)^3^	Intestines (%)^3^	Villus (µm)	Crypt (µm)	V:C^2^ (ratio)
EST													
Control	479^b^	52.2	8.6	43.0	19.4^a^	0.72^b^	0.14	3.2	5.54	4.01	293	69	4.5
Lower	486^a^	52.0	8.2	43.2	19.3^b^	0.79^a^	0.13	3.3	5.45	4.07	328	73	4.5
SEM	1.1	0.08	0.23	0.27	0.07	0.024	0.007	0.25	0.139	0.114	21.3	5.1	0.21
*P*-values													
EST	<0.001	0.12	0.30	0.55	<0.001	0.04	0.60	0.83	0.63	0.73	0.30	0.58	0.97

a-b LSMeans within a column and factor lacking a common superscript differ (*P* < 0.05).

1 8 hatching baskets / treatment (Duration, BW, length: 65 chicks / hatching basket, remaining characteristics: 25 chicks / treatment).

2 RY, residual yolk, YFBM, yolk-free body mass, NAb, IgY natural antibody, stomach = proventriculus + ventriculus, V:C, jejunum villus: crypt ratio.

3 Weight relative to YFBM.

### Chick Quality at Hatch

Lower EST resulted in a 1 mm shorter chick length (*P* < 0.001) and 9.7% higher relative heart weight (*P* = 0.04) compared to control EST (Table 1). Navel score showed an interaction between EST and sex (*P* < 0.001; data not shown). At lower EST, female chicks had worse navel quality with approximately 17.5% higher incidence of navel score 2 or 3 (72.9%) compared to lower EST male chicks and control EST male and female chicks, which were equal to each other (53.6, 57.5, 55.3% score 2+3, respectively). No effect of EST was found for BW, RY, YFBM, NAb IgY, jejunum villus height or crypt depth or V:C, and relative weight of bursa, stomach, or intestines (*P* ≥ 0.12; Table 1).

### Stress Response

No interaction between EST and feeding strategy was found for corticosterone at any age (*P* ≥ 0.17; Table 2). Lower EST resulted in a 6.1 ng / mL higher corticosterone level at hatch (*P* = 0.04) compared to control EST, whereas EST had no effect on corticosterone level at any other age (*P* ≥ 0.44). Early feeding resulted in a 22.3 ng / mL lower corticosterone level at 48 h after hatch (*P* < 0.0001) compared to delayed feeding, whereas feeding strategy had no effect on corticosterone level at any other age (*P* ≥ 0.66).

**Table 2:** Effect of eggshell temperature (EST) during late incubation (≥embryonic d 17; 37.8°C [**control**] or 36.7°C [**lower**]) and/or feeding strategy after hatch (direct access to feed and water [**early**] or 51–54 h deprivation [**delayed]**) on blood corticosterone of broilers at hatch and 48 h, 96 h, and 21 d of age (LSmean ± SEM).

**Treatment**	Corticosterone (ng / mL)^1^			
	Hatch^1^	48 h^2^	96 h^2^	d 21^3^
EST				
Control	21.6^b^	25.0	15.9	6.0
Lower	27.7^a^	27.5	16.5	6.4
SEM	1.94	2.25	2.40	1.31
Feeding strategy				
Delayed	-	37.3^a^	16.2	6.6
Early	-	15.2^b^	16.2	5.8
SEM	-	2.28	2.41	1.31
EST × Feeding strategy				
Control × Delayed	-	35.3	13.4	5.4
Control × Early	-	14.8	18.3	6.6
Lower × Delayed	-	39.4	18.9	7.8
Lower × Early	-	15.6	14.1	5.0
SEM	-	3.19	3.39	1.83
*P*-values				
EST	0.04	0.44	0.85	0.81
Feeding strategy	-	<0.0001	0.99	0.66
EST × Feeding strategy	-	0.61	0.17	0.30

a-b LSMeans within a column and factor lacking a common superscript differ (*P* < 0.05).

1 n = 25 chicks / treatment (divided over 8 hatching baskets / treatment).

2 n = 8 pens / treatment group (2 broilers / pen).

3 n = 8 pens / treatment group (total 100 broilers, unequal nr per pen).

No interaction between EST and feeding strategy or a main effect of EST or feeding strategy was found for any tonic immobility responses (*P* ≥ 0.09; Table 3).

**Table 3:** Effect of eggshell temperature (**EST**) during late incubation (≥embryonic d 17; 37.8°C [**control**] or 36.7°C [**lower**]) and/or feeding strategy after hatch (direct access to feed and water [**early**] or 51–54 h deprivation [**delayed**]) on the number of attempts or vocalizations (mean ± SE) and latency to attempt to stand up (LSmean ±SEM) of broilers during a tonic immobility test at d 13.

**Treatment**	n^1^	Attempts (no.)	Vocalizations (no.)	Latency (sec)
EST				
Control	16	3 ± 0.3	5 ± 2.0	139
Lower	16	2 ± 0.2	6 ± 1.9	170
SEM				27.4
Feeding strategy				
Delayed	16	2 ± 0.2	6 ± 2.1	161
Early	16	3 ± 0.3	5 ± 1.8	148
SEM				27.4
EST × Feeding strategy				
Control × Delayed	8	2 ± 0.4	7 ± 2.7	125
Control × Early	8	3 ± 0.5	4 ± 2.9	154
Lower × Delayed	8	3 ± 0.3	5 ± 3.2	202
Lower × Early	8	2 ± 0.2	6 ± 2.1	143
SEM				38.5
*P*-values				
EST		0.28	0.72	0.62
Feeding strategy		0.69	0.78	0.96
EST × Feeding strategy		0.15	0.09	0.99

1 Pens, with 1 male and 1 female broiler / pen.

### Temperature Preference

No interaction between EST and feeding strategy or a main effect of EST was found for preferred ambient temperature at d 2 or d 12 (*P* ≥ 0.32; Table 4). Early feeding resulted in a 2.2°C and 2.0°C lower preferred ambient temperature at d 2 and d 12, respectively (*P*=0.01 for both d) compared to delayed feeding.

**Table 4:** Effect of eggshell temperature (EST) during late incubation (≥embryonic d 17; 37.8°C [**control**] or 36.7°C [**lower**]) and/or feeding strategy after hatch (direct access to feed and water [**early**] or 51–54 h deprivation [**delayed]**) on preferred ambient temperature of broilers at d 2 or d 12 of age (LSmean ± SEM).

**Treatment**	n^1^	Preferred ambient temperature (°C)	
		d 2	d 12
EST			
Control	8/10	28.3	25.2
Lower	8/10	28.2	25.9
SEM		0.55	0.52
Feeding strategy			
Delayed	8/10	29.4^a^	26.6^a^
Early	8/10	27.2^b^	24.6^b^
SEM		0.55	0.53
EST × Feeding strategy			
Control × Delayed	4/5	29.2	25.9
Control × Early	4/5	27.4	24.5
Lower × Delayed	4/5	29.5	27.3
Lower × Early	4/5	26.9	24.6
SEM		0.78	0.74
*P*-values			
EST		0.85	0.32
Feeding strategy		0.01	0.01
EST × Feeding strategy		0.64	0.40

a-b LSMeans within a column and factor lacking a common superscript differ (*P* < 0.05).

1 Pens at d 2 / d 12, with 2 male and 2 female broilers / pen.

Cloacal temperature linearly increased from d 1 to d 7 (average 40.4 to 41.3 ±0.05°C) and was neither affected by an interaction between EST and feeding strategy (*P* ≥ 0.17; data not shown) nor by a main effect of EST (*P* ≥ 0.12) or feeding strategy (*P* ≥ 0.05).

### Neonatal Development

No interaction between EST and feeding strategy was found for 1st wk mortality (*P* = 0.07), nor for any of the parameters assessed at 48 h after hatch (*P* ≥ 0.05) or for any of the parameters assessed at 96 h after hatch (*P* ≥ 0.28).

**Figure 1:**
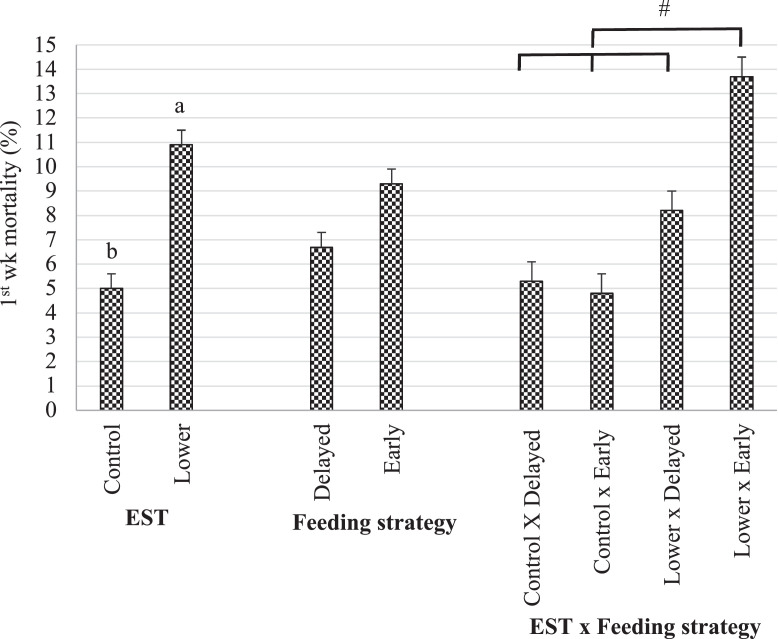
Effect of eggshell temperature (EST) during late incubation (≥embryonic d 17; 37.8°C [**control**] or 36.7°C [**lower**]) and/or feeding strategy after hatch (direct access to feed and water [**early**] or 51–54 h deprivation [**delayed**] on 1st wk mortality of broilers.^a-b^ LSmeans within a factor lacking a common superscript differ (*P* < 0.05). # LSmeans tend to differ (*P* < 0.10). Error bars indicate SEM. n = 8 pens / treatment group (30 broilers / pen).

Lower EST had 5.9% higher 1st wk mortality compared to control EST (*P* < 0.001; Figure 1). At 48 h after hatch, lower EST compared to control EST had higher jejunum V:C (*P* = 0.04; ratio 4.2 vs. 3.9 ±0.11) and higher relative heart weight (*P* = 0.01; 0.92 vs. 0.85 % ±0.017), whereas at 96 h after hatch these differences were not present anymore (*P* ≥ 0.18). No effect of EST on BW, RY, YFBM, relative bursa weight, NAb titer, and jejunum villi length or crypt depth at 48 h or 96 h after hatch (*P* ≥ 0.06; data not shown) was found.

Feeding strategies did not differ in 1st wk mortality (*P* = 0.12; Figure 1). At 48 h and at 96 h after hatch, early feeding compared to delayed feeding had higher BW (48 h: *P* < 0.001; 58.3 vs. 44.4 g ± 0.61/ 96 h: *P* = 0.02; 89.8 vs. 79.5 g ± 2.98), higher YFBM (48 h: *P* < 0.001; 56.1 vs. 42.1 g ± 0.71 / 96 h: *P* = 0.01; 90.0 vs. 78.6 g ± 3.02), and longer jejunum villi length (48 h: *P*<0.01; 420 vs. 375 µm ± 8.8 / 96 h: *P* = 0.01; 497 vs. 429 µm ± 16.8). At 48 h after hatch, early feeding compared to delayed feeding had deeper jejunum crypt (*P* < 0.001; 109 vs. 91 µm ± 2.3) and lower IgY NAb titer (*P* = 0.04; titer 3.8 vs. 4.4 ± 1.7), whereas at 96 h after hatch these differences were not present anymore (*P* ≥ 0.44). Feeding strategy had no effect on RY, relative heart weight, jejunum V:C, or relative bursa weight at 48 h or 96 h after hatch (*P* ≥ 0.06; data not shown).

## DISCUSSION

We hypothesized that delayed access to feed and water after hatch would be stressful for chicks, leading to elevated corticosterone levels in the short term and resulting in higher fearfulness at a later age. Furthermore, delayed feeding was expected to result in higher preferred ambient temperatures during rearing and a low EST during late incubation was suggested to interact with post-hatch feeding strategy on temperature preference as well as neonatal broiler development in general. First, the effects of feeding strategy will be discussed, followed by whether EST interacts with feeding strategy, and finally, the main effects of EST.

### Feeding Strategy

At 48 h after hatch, 2.5 times higher corticosterone levels were found in 48 h delayed fed broilers compared to early fed broilers. The higher corticosterone level found in delayed fed broilers was likely the result of exposure to stress due to feed and water deprivation. Corticosterone level increased between hatch and 48 h after hatch in delayed fed broilers, whereas in early fed broilers it decreased during that time span. Moreover, once delayed fed broilers had access to feed and water for 48 h, their corticosterone level reduced to levels similar to those of early fed broilers. Our finding is in line with Van de Ven et al. (2013) who showed that chicks that hatched in a Patio hatching system with direct access to feed and water had lower corticosterone concentration at pull compared to chicks that hatched in a conventional hatcher without feed and water.

The increase in corticosterone in delayed fed broilers could also be in response to a lack of glucose. Delayed fed broilers likely had limited glucose available during the first 48 h after hatch, since most glycogen storages are used during hatching (Freeman, 1965; Christensen et al., 2001; Molenaar et al., 2013; van de Ven et al., 2013; Maatjens et al., 2014) and no exogenous glucose via the diet was provided. Blood glucose was not determined in the current study, but it has previously been shown that 48 h delayed fed broilers had lower blood glucose concentration after hatch compared to early fed conspecifics (Gaglo-Disse et al., 2010; Wang et al., 2014; Khosravinia & Manafi, 2016). Corticosterone level may be increased because corticosterone increases gluconeogenesis by a decrease in the uptake of glucose by muscle cells and an increase in protein degradation for the supply of glucogenic amino acids (Mench, 2002). Consequently, in delayed fed broilers, corticosterone levels may rise, to maintain glucose homeostasis. However, it is unclear whether this mechanism occur independent of stress or whether it is also part of the stress response. In stressful situations, there is a higher demand for directly accessible energy, such as glucose, for a fight or flight response. Furthermore, there may not be a long-lasting association between corticosterone and glucose concentrations because blood glucose concentration can remain low during 2 wk post hatch after delayed feeding (Gaglo-Disse et al., 2010), whereas corticosterone level does not. Studies on early feeding that measured additional stress indicators next to corticosterone are rare. Khosravinia and Manafi (2016) found that unfed day old chicks showed decreasing resting behaviour, and increasing active wakefulness and attempts to escape from a box without feed and water as time progressed. The aforementioned findings support that the higher corticosterone that was found in the current study at 48 h after hatch in delayed fed broilers are due to a stress response rather than for glucose homeostasis.

We hypothesized that exposure to stress during early life due to delayed feeding would result in higher fearfulness at a later age. However, no indications that early and delayed fed broilers differed in fearfulness were found in the current study, as no difference was present during a tonic immobility test at d 13. Besides, corticosterone level at d 21 was similar between both feeding strategies. It should be noted that corticosterone at d 21 was determined without applying a stressor. Hedlund et al. (2019) showed in laying hens that although chicks experienced more stress during hatchery processes, their baseline corticosterone levels at d 6 or 41 did not differ, however following a tonic immobility test, corticosterone levels increased to a larger extent in hens that experienced more stress during early life despite fearful behavior being comparable. This suggests that feeding strategy during early life possibly affects stress responsiveness during later life when exposed to certain stressful conditions, even though baseline corticosterone level and behavioral response to a tonic immobility test did not differ in the current study.

Feeding strategy evidently affected preferred ambient temperature during rearing. Feeding increases thermogenesis by digestion of feed and body growth and early fed broilers produced approximately twice as much heat at pulling compared to delayed fed broilers (van Roovert-Reijrink et al., 2017). In the current study, early fed broilers had higher BW, YFBM, and longer jejunum villi length compared to delayed fed broilers up to at least 96 h after hatch. This supports the idea that early fed broilers are ahead in body growth and development, and suggests that thermogenesis is indeed higher in early fed broilers. On the one hand, higher thermogenesis seems to beneficially affect cold tolerance. Early fed broilers showed milder drops in rectal temperature after cold exposure (20°C) at d 2 to 3 compared to delayed fed broilers (Van de Brand et al., 2010). On the other hand, higher thermogenesis could increase the risk of heat stress in early fed broilers. At d 2 after hatch, the delayed fed broilers preferred an ambient temperature of 29.4°C, which is in line with commercial recommendations for whole-house brooding temperature (Aviagen, 2018). At the same age, early fed broilers preferred an ambient brooding temperature of 27.2°C. In current recommended whole house brooding temperature setpoints no distinction is made between early and delayed fed broilers. Incorrect ambient brooding temperatures can negatively impact growth performance and increase ascites-related mortality (Deaton et al., 1996; Lourens et al., 2005; Leksrisompong et al., 2009; Van der Pol et al., 2013). Strikingly, the difference in preferred ambient temperature between early and delayed fed broilers was still present at d 12, which suggests that feeding strategy post hatch may have long-lasting effects on thermoregulation.

### Incubation Temperature × Feeding Strategy

It was expected that early feeding would result in a lower temperature preference compared to delayed feeding when incubated at a control EST of 37.8°C, whereas this would not be the case when EST is lowered during late incubation, due to a lower EST of 36.7°C during late incubation resulting in a higher preferred ambient temperature at d 1 (Wijnen et al., 2020). In the current study, no effect of lower EST on temperature preference was found and consequently, the hypothesis of an interaction with feeding strategy was rejected.

De Jong et al. (2017) suggested that the effects of early feeding on neonatal chick development may be dependent on incubation temperature. We hypothesized that the need for early feed intake would be higher in chicks incubated at a lower EST of 36.7°C during late incubation because it was previously shown that chicks incubated at this lower EST have less RY available relative to YFBM at hatch moment (Maatjens et al., 2016). In the current study, no differences in YFBM and RY were found between a lower EST of 36.7°C during late incubation and a constant 37.8°C EST. This may explain why no interaction between EST and feeding strategy was found for any of the neonatal broiler development parameters.

Regardless of treatment group, 1st wk mortality was high (an average of 7.7%). This was probably the result of a bacterial infection because necropsy showed that most broilers (approx. 70%) had ecolisepticemia, yolk sac infection, or both. The old parental flock (54 wk) and lack of disinfection of hatching eggs, drinking water, and hatcher, may have increased the bacterial load (Yassin et al., 2009; Fertner et al., 2011; Poulsen et al., 2017). Eggshell temperature during late incubation tended to interact with feeding strategy on 1st wk mortality. First wk mortality tended (*P* = 0.07) to be higher in the lower EST with early feeding group (13.7%) compared to the other 3 treatment groups (average 6.1%). The biological mechanism explaining this tendency is unclear. It could be that early fed broilers were exposed to a relatively larger bacterial load and at an earlier age, thus enabling bacteria to replicate and be spread via feces and drinking water. While this does not appear to be problematic in good quality chicks, in chicks with lower quality navels, such as those in lower EST incubated female chicks, this might lead to higher mortality. A badly closed navel increases the risk of yolk sac infection and 1st wk mortality (Brandly, 1932; Dardiri et al., 1955; Fasenko and O'Dea, 2008; Olsen et al., 2012). This may also explain the higher mortality rate that was found after early feeding in another study (Lamot et al., 2016), whereas early feeding generally lowers mortality rates (de Jong et al., 2017). Yet, it should be emphasized that these are speculations based on a tendency for 1st wk mortality. Moreover, 1st wk mortality was similar for both sexes, whereas only females had worse navel conditions. Future studies on a potential relationship between incubation temperature and post hatch feeding strategy are required.

### Incubation Temperature

The finding that lower EST incubated chicks had higher corticosterone level at hatch compared to control EST indicated that lower EST may have caused cold stress to the embryo. Late-term embryos are capable of showing stress responses because the hypothalamic-pituitary-adrenal axis becomes functional at around d 14 of incubation (Case, 1952; McIlhone, 2011) and corticosterone level at hatch can be altered by thermal manipulations during incubation (Moraes et al., 2002; Amjadian and Shahir, 2020). Although avian embryos act poikilotherm during the majority of incubation time and full thermoregulatory response develops mainly after hatching (Dietz and van Kampen, 1994; French, 1997), embryos do decrease blood flow to the chorioallantois and increase heat production when eggs are cooled by 1°C or more (Romijn and Lonkhorst, 1955; Tazawa et al., 1988; Tazawa et al., 1989; Nichelman and Tzschentke, 1999; Lourens et al., 2006; Szdzuy et al., 2008). However, in the current study, these abilities appear to be insufficient, resulting in signs of cold stress when lowering EST during late incubation.

In conclusion, a lower EST during late incubation as well as delayed feed access after hatch appears to be stressful perinatal conditions. Also, early post hatch feeding strategy can have a long-lasting programming effect on thermoregulation and consequently on preferred ambient temperature during rearing.

## Appendix Supplementary materials

Image, application 1

## Footnotes

Supplementary material associated with this article can be found in the online version at doi:10.1016/j.psj.2022.102088.
