# Supplementary material for: Effects of late incubation temperature and moment of first post-hatch feed access on neonatal broiler development, temperature preference, and stress response
Source: Poult Sci. 2022 Aug 3;101(10):102088. doi: 10.1016/j.psj.2022.102088 (PMC9449862; doi:10.1016/j.psj.2022.102088)
Supplement: Supplementary file 1 [file mmc1.docx]

**Figure S1**. Actual average, minimum, and maximum eggshell temperature (**EST**) during embryonic d (**E**) 17 until E19 12h of broiler eggs incubated either A) at a setpoint of 37.8°C EST or B) at a setpoint of 36.7°C EST.
